# Evolving Cancer Characteristics Among World Trade Center Survivors: An Updated Analysis from the WTC Environmental Health Center

**DOI:** 10.3390/ijerph23050625

**Published:** 2026-05-08

**Authors:** Nedim Durmus, Ziyue Wang, Alan A. Arslan, Emre Goren, Ramazan Alptekin, Yujia Lou, Andrew Shao, Nida Athar, Yibeltal A. Ashebir, Yidan Shi, Leigh Wilson, Joan Reibman, Yongzhao Shao

**Affiliations:** 1 Division of Pulmonary Medicine, Department of Medicine, New York University Grossman School of Medicine, New York, NY 10016, USA; joan.reibman@nyulangone.org; 2 World Trade Center Environmental Health Center, NYC Health + Hospitals, New York, NY 10016, USA; emre.goren@nyulangone.org (E.G.); dr.r.alptekin@gmail.com (R.A.); andrewshao01@gmail.com (A.S.); leigh.wilson@nyulangone.org (L.W.); 3 Department of Population Health, New York University Grossman School of Medicine, New York, NY 10016, USA; ziyue.wang@nyulangone.org (Z.W.); alan.arslan@nyulangone.org (A.A.A.); camila.lou@nyulangone.org (Y.L.); nida.athar@nyulangone.org (N.A.); yibeltal.ashebir@nyulangone.org (Y.A.A.);; 4 Department of Obstetrics and Gynecology, New York University Grossman School of Medicine, New York, NY 10016, USA; 5 Department of Biostatistics, New York University School of Global Public Health, New York, NY 10003, USA

**Keywords:** World Trade Center, environmental exposure, WTC survivors, cancer, cancer characteristics

## Abstract

**Highlights:**

**Public health relevance—How does this work relate to a public health issue?**

- World Trade Center (WTC) survivors were exposed to complex mixtures of carcino genic environmental agents following the 9/11 disaster, representing a unique large-scale environmental health event.
- This study characterizes the evolving cancer burden in a diverse civilian population with WTC exposure, addressing an important gap beyond responder-focused research.

**Public health significance—Why is this work of significance to public health?**

- The number of certified cancers among WTC survivors has increased 2.5-fold over the last five years, highlighting a major cancer burden 25 years after 9/11.
- Distinct cancer patterns, including elevated frequencies of melanoma of the skin and urinary bladder cancer, underscore the need to understand exposure-related and surveillance-related influences in this WTC affected urban civilian population.

**Public health implications—What are the key implications or messages for practitioners, policy makers, and/or researchers in public health?**

- Evidence-based strategies for sustained surveillance and targeted cancer screening are essential for high-quality healthcare and research in an aging population exposed to environmental disasters.
- Study findings support the need for continued epidemiologic and mechanistic research to clarify exposure–cancer risk and outcomes relationships and inform future public health policy.

**Abstract:**

Local community populations (“survivors”) exposed to the World Trade Center (WTC) disaster experienced complex exposures to mixtures of dust and combustion products with potential carcinogenic effects. Survivors with certifiable WTC-related conditions are eligible for inclusion in the federally funded WTC Health Program. We provide an updated description of cancers in the WTC Environmental Health Center (EHC), a program for WTC survivors, through 31 December 2024. Using data from the WTC EHC Pan Cancer Database, we summarized demographics, exposure history, and tumor characteristics among enrollees with pathologically confirmed primary cancers meeting WTC Health Program certification criteria. Among 17,449 members, 7274 had a certifiable cancer diagnosis; excluding non-melanoma skin cancers, 6588 patients with 7643 eligible cancers were analyzed. Women comprised 50.3% of the cohort and 47.5% of diagnoses. Solid tumors accounted for 87% of certifications, with breast (22%) and prostate (19%) cancers most frequent, followed by lung (8%), thyroid (6%), colorectal (6%), and melanoma (4.5%). Lymphoproliferative and hematopoietic malignancies represented 13% of cases. Fourteen percent developed multiple primary cancers, and median latency clustered around 14–16 years. Compared with our previous report in 2020, the enrolled number of cancers increased 2.5-fold. These findings support the need for sustained surveillance and additional epidemiologic studies to improve cancer prevention and survivorship in this uniquely exposed population.

## 1. Introduction

The destruction of the World Trade Center (WTC) towers on 11 September 2001 released large quantities of aerosolized dust and smoke that affected the surrounding community [1,2,3]. Community members (WTC survivors), including local residents, local workers, students, those in the disaster area on 11 September 2001, and individuals involved in cleaning contaminated sites, had potential for acute exposures from the dust clouds generated by the collapsing buildings and/or chronic exposures to resuspended dust and fumes from fires that burned through December 2001 [4,5,6,7]. The dust and fumes contained respirable particulate matter composed of pulverized cement, glass fibers, asbestos, lead, and combustion products, together with complex mixtures of volatile and semi-volatile chemicals, including polycyclic aromatic hydrocarbons, polychlorinated biphenyls, and polychlorinated furans and dioxins [2,8,9,10,11,12,13,14,15,16,17,18,19].

The WTC Environmental Health Center (WTC EHC) was established in the aftermath of 9/11 and became a Clinical Center of Excellence (CCE) for community members (“survivors”) within the WTC Health Program (WTC Health Program) created under the James Zadroga 9/11 Health and Compensation Act of 2010 [4,20]. Enrollment and inclusion in the WTC EHC require defined WTC exposure and a physical and/or mental health condition certified by the federal government according to published rules including defined certifiable cancers with specified cutoffs for latency periods [20,21].

Overall cancer rates have been described in WTC-exposed firefighters and responders that are 6–14% higher than background rates [22,23,24,25]. Specifically, excess rates of prostate, thyroid, skin, and tonsil cancers have been described [23,24,26,27,28,29,30]. Recent studies report exposure–response relationships [19,20,21,22,23,24,25,26,27,28,29,30,31,32]. Survivor populations differ from those of the responder populations, with more women (50%), diverse age/ethnicity, and age distribution [4,20,33]. Fewer studies are available for cancer incidence rates in this population; however, the New York City WTC Health Registry reported an all-cancer SIR of 1.08 in non-rescue and recovery workers through 2011, with elevated rates of breast, prostate, melanoma, and thyroid cancers in this population [34,35].

The survivor program in the WTC Health Program and the WTC EHC also differ from the programs for rescue and recovery workers, as survivors must have WTC exposure and a certifiable condition for inclusion. This self-referred group thus precludes simple analysis of cancer incidence. We have therefore reported descriptions of the observed cancers in this population to understand the distribution and characteristics of cancers. We previously described 2999 cancers in 2561 patients as of December 2019 [36,37] and subsequently reported characteristics of lung cancer, including that in women, breast cancer, mesothelioma, as well as cancers in those exposed to the WTC disaster at a young age [37,38,39,40]. Characteristics of survivors enrolled in the WTC Health Program including the national program, have also been described [20].

The WTC-associated cancer burden continues to rise after more than two decades, underscoring the need for sustained surveillance and refined characterization of cancers in this population. We present an updated and expanded description of cancers including those identified over the past 5 years in the WTC EHC and captured in the WTC EHC Pan-Cancer Database (PCDB) through 31 December 2024 [36,37].

## 2. Materials and Methods

### 2.1. WTC EHC and Study Participants

The WTC EHC was created in response to community requests in the years after 9/11 and was eventually included as the Center of Excellence in the WTC Health Program for local community members, subsequently called “survivors” [4,5,20]. Patients self-refer to this program and must have exposure as a local resident, local worker, student, or passer-by on 9/11 and the presence of a “Certifiable condition” such as a WTC-related aerodigestive disorder or cancer [22]. All ages, including those in utero on 9/11, are eligible. The WTC EHC conducts continuous follow-up, with invitations for routine clinical evaluations every 12–18 months. At each visit, participants undergo standardized physical and mental health assessments including the capture of any new cancer diagnoses. All cancer diagnoses are independently verified with review of pathology or cytology reports, and all cancer characteristics are entered in the WTC EHC PCDB [36,37].

For this analysis, we included patients with a diagnosis of cancer who were enrolled in the WTC EHC as of 31 December 2024 and had a cancer diagnosis according to the latency rules defined by the WTC Health Program. We excluded non- melanoma skin cancers due to their known inconsistent reporting and underreporting in national and state tumor registries compared to other malignancies. A cancer is considered WTC-related based on cancer type, exposures, and minimum latencies for the categories of cancer defined by the WTC Health Program on 17 October 2012 and revised subsqequently [21,41]. Briefly, minimum latency for inclusion as a WTC related cancer is as follows: (1) mesothelioma—11 years, (2) all solid cancers (other than mesothelioma, lymphoproliferative, thyroid, and childhood cancers)—4 years, (3) lymphoproliferative and hematopoietic cancers—0.4 years, (4) thyroid cancer—2.5 years, and (5) childhood cancers (other than lymphoproliferative and hematopoietic cancers)—1 year [21]. Effective 18 January 2023, the WTC Health Program added all types of uterine cancers, including endometrial cancer, as certifiable conditions [42].

We also included WTC EHC participants without a certified cancer diagnosis who were enrolled at WTC EHC clinical sites during the same period as a non-cancer group. The comparison to non-cancer participants was intended to provide context regarding the demographic and exposure characteristics of individuals within the WTC EHC population.

This study was approved by the New York University School of Medicine Institutional Review Board (IRB). Reference groups of non-cancer participants and cancer patients were analyzed after removal of personal identifiers with IRB approval to review de-identified data (IRB number: i06-1).

### 2.2. Exposure Assessment

The WTC Health Program determines an applicant’s eligibility for an initial health evaluation based on one of the following criteria: (1) The screening applicant was present in the dust or dust cloud in the New York City disaster area on 11 September 2001. (2) The screening applicant worked, resided, or attended school, childcare, or adult daycare in the New York City disaster area for at least (a) 4 days during the period beginning on 11 September 2001 and ending on 10 January 2002 or (b) 30 days during the period beginning on 11 September 2001 and ending on 31 July 2002. (3) The screening applicant worked as a cleanup worker or performed maintenance work in the New York City disaster area during the period beginning on 11 September 2001 and ending on 10 January 2002 and had extensive exposure to WTC dust as a result of such work. (4) The screening applicant (a) was deemed eligible to receive a grant from the Lower Manhattan Development Corporation Residential Grant Program, (b) possessed a lease for a residence or purchased a residence in the New York City disaster area, and (c) resided in such residence during the period beginning on 11 September 2001 and ending on 31 May 2003. (5) The screening applicant is an individual whose place of employment (a) at any time during the period beginning on 11 September 2001 and ending on 31 May 2003 was in the New York City disaster area and (b) was deemed eligible to receive a grant from the Lower Manhattan Development Corporation WTC Small Firms Attraction and Retention Act program or other government incentive program designed to revitalize the lower Manhattan economy after the 11 September 2001 terrorist attacks [42,43].

Survivors who have been determined to have screening-eligible status under the above rules may seek status as a certified-eligible survivor based on a certification by the WTC Health Program that, pursuant to an initial health evaluation, the screening-eligible survivor has a WTC-related health condition and is eligible for follow-up monitoring and treatment.

Baseline and follow-up questionnaires capture key exposures, including WTC-related, occupational, and lifestyle factors (e.g., smoking). Acute dust cloud exposure and other WTC-related exposures are self-reported at the time of initial enrollment into the WTC Environmental Health Center. Enrollment began in 2005 and is ongoing.

### 2.3. WTC Pan Cancer Database

The WTC EHC PCDB maintains information about “Certified Cancers” in the WTC EHC Survivors. Only primary cancer diagnoses confirmed with a pathology/cytology report and/or cancers reported for the WTC EHC participants from Tumor Registries of New Jersey, New York and Pennsylvania are included in this report. These cancer registries have data exchange agreements with many other states so that residents diagnosed or temporarily living elsewhere would be included. The International Classification of Diseases for Oncology (ICD-O-3) was used to classify cancers [44]. The demographic information, cancer characteristics, and cancer biomarker information are included in the database as previously reported [36,37].

### 2.4. Statistical Analysis

Summary statistics were presented for WTC exposure and patient demographic characteristics. Categorical variables were summarized with counts and proportions, and continuous variables with median and interquartile range (IQR). Comparisons between WTC cancer and non-cancer groups were conducted using the Wilcoxon rank-sum test for continuous variables and Chi-square test for categorical variables. Two-sided *p*-values < 0.05 were considered statistically significant. For cancer-specific analyses, we described the distribution of certified primary cancer diagnoses overall and by sex, including the frequency and proportion of each cancer type, the number of primary cancers per patient, the median (and IQR) age at first cancer diagnosis, and the median (and IQR) latency time in years from 11 September 2001 to first cancer diagnosis. The open-source statistical software R-4.4.3 was utilized to conduct these analyses.

## 3. Results

### 3.1. Participants

**Figure 1:**
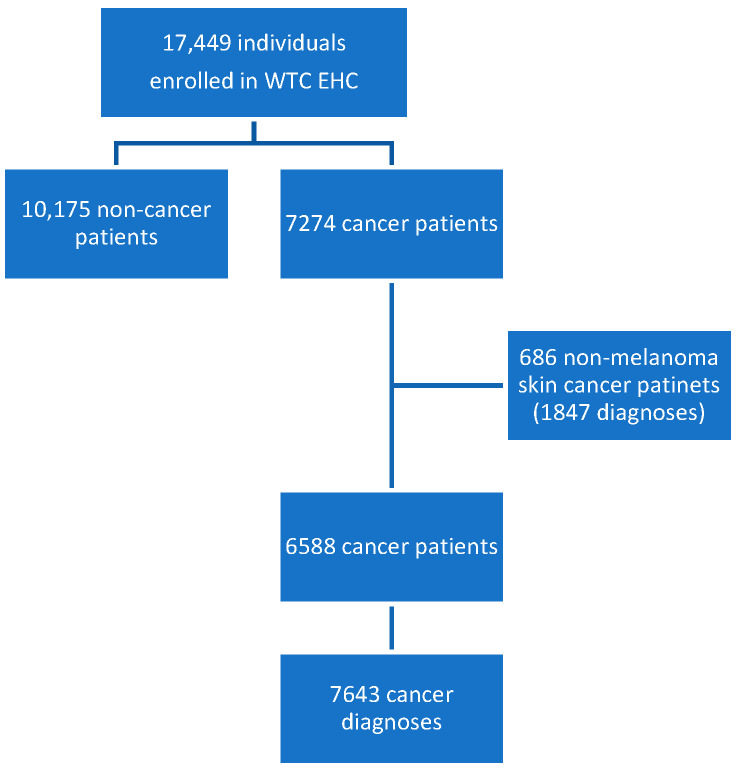
WTC EHC patients included for cancer analysis as of 31 December 2024.

We identified 17,449 individuals included in the WTC EHC as of 31 December 2024. Of these, 7274 individuals had a diagnosis of cancer and 10,175 had certifiable conditions other than cancer. For this analysis, we excluded non-melanoma skin cancers and patients with non-melanoma skin cancer (n = 686), resulting in 6588 cancer patients with 7643 WTC-certified cancer diagnoses (Figure 1).

Table 1 presents enrollment characteristics for 16,763 WTC EHC participants (10,175 without cancer and 6588 with cancer). Women comprised 50.3% of the overall population and 47.5% of the cancer patients. Median age on 11 September 2001 was 41 and 45 for non-cancer and cancer patients, respectively. In the cancer group, Non-Hispanic White participants represented a larger proportion than in the non-cancer group (46.0% vs. 35.7%), whereas Hispanic participants were less represented in the cancer compared to non-cancer group (11.0% vs. 24.6%). Body mass index categories and indicators of socioeconomic status were comparable between non-cancer and cancer groups. Acute WTC dust cloud exposure metrics were similar across groups; about half reported being caught in the 9/11 dust cloud (cancer 49.7%, non-cancer 53.0). Never-smokers predominated in both groups; however, former smoking was more frequent among cancer patients (31.8% vs. 25.9%), and a greater proportion of the cancer cohort reported >5 pack-year tobacco use (33.2% vs. 24.9%) (Table 1).

**Figure 2:**
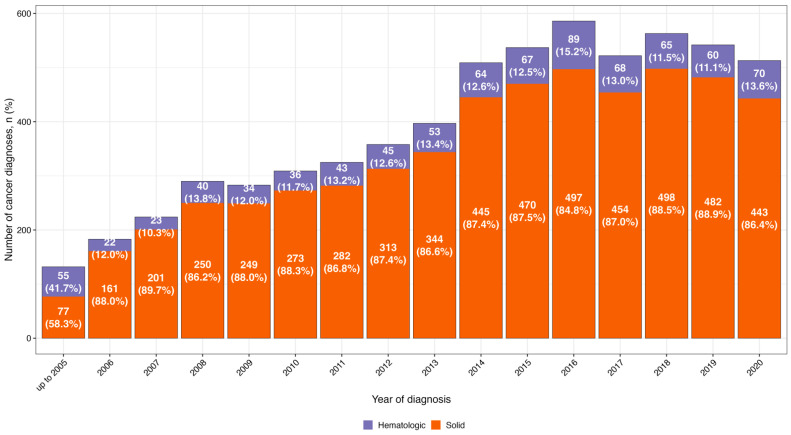
Distribution of the percentage of the solid and lymphoproliferative/hematopoietic malignancies per year in the WTC EHC through 31 December 2020.

The percentage and the number of the solid and lymphoproliferative/hematopoietic cancer diagnoses in the WTC EHC per year through 31 December 2020 are shown in Figure 2. The number of both solid and lymphoproliferative/hematopoietic cancer diagnoses increased each year, but the percentage distribution of solid and lymphoproliferative/hematopoietic cancers were very similar each year. We expect the distribution in Figure 2 to be stable before 2018, while the count of yearly cancers after 2020 are unstable thus later years are not shown in Figure 2, and the count of yearly cancers between 2018 and 2020 may slightly change by time for several reasons. First, there are a few years of delays in obtaining cancer data from state registries. Second, there are a few years of delays for cancer patients to be certified by CDC and finally eligible to be enrolled to our cohort and input into the pan-cancer database. Third, after 2018, eligible cancer patients from the local community can either enroll in our cohort or enroll in the new Williams Street Clinic (WSC), which opened in August 2018. We currently do not have the yearly cancer diagnosis data from WSC and cannot have a very reliable count of yearly cancer diagnosis after 2018. As of December 31, 2024, we identified a total of 6588 patients with 7643 cancer diagnoses with the overall distribution of solid (n = 6665, 87%) and lymphoproliferative/hematopoietic cancer diagnoses (n = 978, 13%) (Figure 2).

The data for the distribution of the percentage of the solid and lymphoproliferative/hematopoietic malignancies in the WTC EHC through 31 December 2020 has also been shown in Supplemental Table S2.

### 3.2. Cancer Distribution

**Figure 3:**
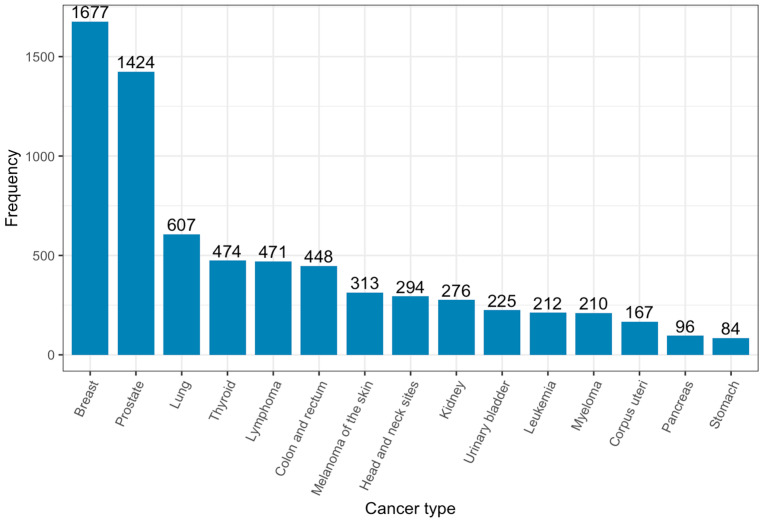
Frequency of top fifteen cancer diagnoses as of 31 December 2024.

The fifteen most common cancer diagnoses are shown for the whole population as of 31 December 2024 (Figure 3). Overall, breast (n = 1677, 22%) and prostate (n = 1424, 19%) cancer diagnoses were the most common cancers, followed by lung (n = 607, 8%), thyroid (n = 474, 6%), and lymphoma (n = 471, 6%) (Figure 3). Rare cancers were also identified in the population. These included male breast cancer (n = 22), adenoid cystic carcinoma (n = 11), head and neck cancers (n = 9), thymic carcinoma (n = 11), gallbladder cancer (n = 8), and mesothelioma (n = 6).

### 3.3. Distribution of Solid Cancer Diagnoses in Female and Male Patients

**Figure 4:**
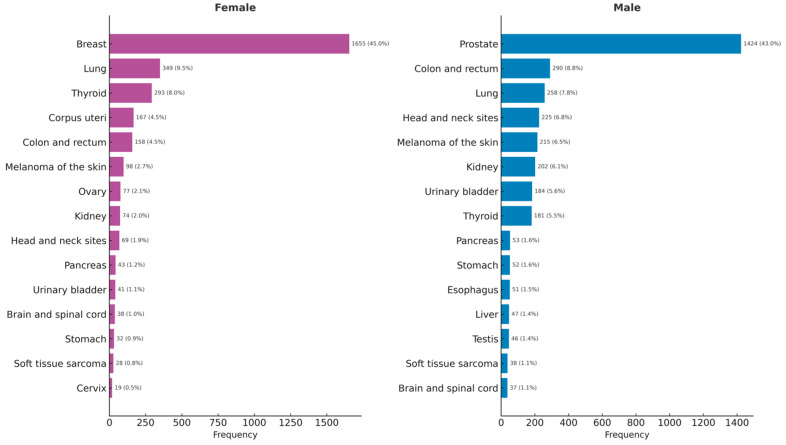
Frequency of top fifteen solid cancer diagnoses in female and male patients.

Among women, breast cancer accounted for half of all solid cancer diagnoses (50%), with lung (11%), thyroid (9%), uterine (5%), and colon/rectum (5%) cancers following. In men, prostate cancer predominated (39%), with subsequent frequencies for colon/rectum (8%), lung (7%), head and neck (6%), and melanoma of the skin (6%) cancers (Figure 4).

### 3.4. Age of Cancer and Latency from 11 September 2001

The median age in years at first cancer diagnosis and median latency in years from 9/11 for the top fifteen cancer types are shown for the total population as well as for female and male patients (Table 2). The median age and latency in years for less common cancer types are shown in Supplemental Table S1, and the cancers which have less than 10 diagnoses were excluded.

### 3.5. Distribution of Lymphoproliferative and Hematopoietic Malignancies

**Figure 5:**
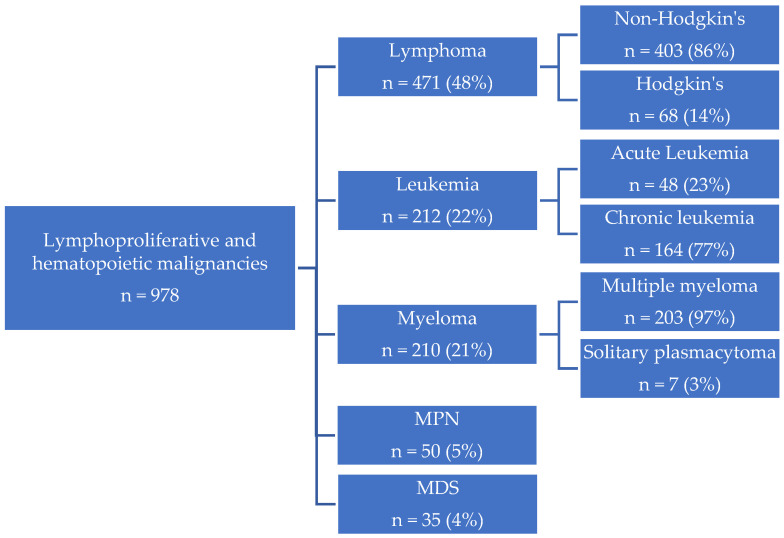
Distribution of lymphoproliferative and hematopoietic malignancies in the WTC EHC (MPN = myeloproliferative neoplasms (MPN); MDS = myelodysplastic syndromes).

Among lymphoproliferative and hematopoietic malignancies, lymphomas comprised the largest share (n = 471; 48%), followed by leukemias (n = 212; 22%), myelomas (n = 210; 21%), myeloproliferative neoplasms (MPN) (n = 50; 5%), and myelodysplastic syndromes (MDS) (n = 35; 4%). Within lymphomas, non-Hodgkin’s lymphoma predominated (n = 403; 86%), with Hodgkin’s lymphoma diagnosed in 68 patients (14%). Acute leukemia was identified in 23% (n = 48), and 77% were chronic leukemias. Nearly all myelomas were multiple myeloma (n = 203; 97%), with solitary plasmacytoma representing 3% (n = 7) (Figure 5).

### 3.6. Cancer Multiplicity

Among the 6588 cancer patients, 944 (14%) had a diagnosis of more than one cancer, with 842 patients (12.8%) with two primary cancers and 102 patients with three or more primary cancers (Table 3). The percentage distribution was similar in 2019 and 2024 (Table 3).

We performed a stratified analysis of the patients with multiple cancers according to sex, age at diagnosis of first cancer, and smoking status. The distribution of the multiplicity in female and male and age at diagnosis under the age of 50 and over 50 were similar. The percentage of the multiple cancers in the ever smokers was slightly higher compared with never smokers (Table 4).

## 4. Discussion

We updated and expanded the characterization of cancers in the WTC EHC, reporting cancers identified over a 24-year period since 11 September 2001. Since the previous report in December 2019 [35], which described 2561 patients with certified cancers excluding non-melanoma skin cancer, the number of patients with certified cancer enrolled in the WTC EHC has increased approximately 2.5-fold. These findings raise important questions regarding the association between World Trade Center exposure and cancer development. However, this study is descriptive and does not allow assessment of causal relationships or quantification of associations between WTC dust exposure and cancer development.

Several factors may contribute to the described increase in the number of patients with cancer enrolled in the WTC EHC. Enrollment of survivors in the WTC EHC is based on self-referral, and, importantly, this data does not represent cancer incidence in the underlying exposed population. However, the increase in patients in the program is notable. One potential reason for the increase may be due to changes in referral patterns with increased awareness of and referrals to the program contributing to higher enrollment. Some individuals may have had cancers diagnosed prior to the enrollment, with delayed enrollment related to administrative barriers or limited awareness of the program. In addition, the aging of the cohort is likely associated with an increased risk of cancer leading to increased enrollment. The WTC Health Program provides and promotes cancer screening and conducts routine laboratory testing and imaging, which may facilitate earlier detection of malignancies. This process may enhance earlier detection, however many cancers among enrollees were diagnosed at outside institutions before referral to the program. Finally, the observed increase may reflect cancer latency from an environmental exposure, as carcinogenesis is a multistep process involving the accumulation of genetic alterations and changes in the tumor microenvironment over time. Consequently, cancers may not become clinically apparent until many years after exposure. These potential explanations warrant further investigation. We also displayed the overall yearly cancer diagnosis data in Figure 2; however, the data needs to be interpreted with caution. We expect the distribution and increasing trend to be reliable before 2018, while the yearly counts of cancers after 2020 are unstable. We also expect the count of yearly cancers between 2018 and 2020 may slightly change by time for several reasons. First, there are a few years of delays in our process of obtaining cancer data from state registries. Second, there are a few years of delays for cancer patients to be certified by CDC and finally eligible to be enrolled to our cohort and be input into the pan-cancer database. Third, after 2018, eligible cancer patients from local community can either enroll in our cohort or enroll in the new Williams Street Clinic (WSC), which opened in August 2018. We currently do not have the yearly cancer diagnosis data from WSC and cannot determine a very reliable count of yearly cancer diagnosis after 2020 in Figure 2.

The distribution of solid and hematopoietic malignancies was similar to that reported previously [36]. The distribution of cancer pattern is also consistent with findings from a recent study of WTC survivors enrolled in the national program, in which colorectal and lung cancers were the most frequently diagnosed malignancies [20]. We also describe rare cancers. Despite the increase in the number of cancer diagnoses, the distribution of multiple primary cancers was similar to that reported in the initial 2019 analysis [36]. Latency periods clustered around 14 to 16 years and smoking patterns were similar across groups, although former smoking and higher cumulative smoking exposure were more common among patients with cancer. However, melanoma of the skin and urinary bladder cancer ranked higher among the 15 most common cancers in both women and men compared with the previous report [36]. Notably, cutaneous melanoma emerged as one of the most common solid tumors in both women and men, ranking sixth and fifth, respectively. This represents a marked change from earlier analyses, in which melanoma ranked eleventh among women and tenth among men, and differs from distributions reported in the national WTC Health Program [20]. The increase in melanoma diagnoses occurred over a relatively short period. Elevated incidence of cutaneous melanoma has been reported among non-Hispanic White participants in the WTC Combined Rescue/Recovery Cohort compared with the New York State general population, with evidence of a dose–response association by arrival time at the WTC site [45]. Collectively, the increase in melanoma among both women and men enrolled in the WTC EHC warrants further investigation, particularly given the established role of environmental exposures in melanoma.

Women comprised approximately half of the study population and as such, this study highlights the importance of malignancies in this population. Most malignancies were sex-specific solid tumors, with breast and prostate cancers representing the most common cancers similar to the general population. Breast cancer was the most frequently diagnosed cancer among women enrolled in the WTC EHC. Although breast cancer is the most common cancer in women in the Surveillance, Epidemiology, and End Results (SEER) Program, the age at diagnosis for this cancer in the WTC EHC was younger than median ages reported in the SEER data in 2021 [46]. Prior analyses described the clinicopathologic characteristics of breast cancer among WTC-exposed survivor women [47]. In those studies, a higher proportion of grade 3 (poorly differentiated) tumors and a higher prevalence of triple-negative breast cancer among Hispanic women were observed compared with SEER-18 data. Since the prior report, the number of breast cancer diagnoses among survivor women in the WTC EHC has nearly tripled. Uterine cancer has also emerged among the 15 most frequently diagnosed cancers in women; however, this change may be related to revisions in cancer certification criteria implemented by the National Institute for Occupational Safety and Health effective 18 January 2023 [42]. Importantly, lung cancer remains among the top three most frequently diagnosed cancers in both women and men, with a slightly younger age at diagnosis than reported in SEER data [47]. Previous analyses of the WTC EHC cohort described a substantial proportion of lung cancer cases among women, with a younger age at diagnosis compared to SEER data and a predominance of adenocarcinoma and carcinoid histologic subtypes [39]. We described lung adenocarcinoma in women with molecular alterations including *EGFR*, *ALK*, *KRAS*, *ROS1*, and *BRAF* mutations and identified a relatively high proportion of women never-smokers with lung cancer in this population [48]. Recently, a dose–response relationship to WTC particulate dust or debris and lung cancer incidence among WTC responders was reported [34]. Collectively, these findings underscore the need for further study of the survivor female population.

There are potential exposure-related molecular pathways that may contribute to cancers in this population. Recent studies from our group demonstrated long-term epigenetic alterations in blood samples from community members exposed to the WTC disaster, with enrichment of cancer-related pathways and potential epigenetic links between WTC exposure and breast cancer [49,50,51,52,53,54]. Experimental and molecular studies suggest that inhalational exposure to World Trade Center dust can disrupt gene expression and immune cell infiltration in the prostate, with persistent alterations observed up to 30 days after exposure in animal models [55]. Long-term epigenetic changes associated with WTC dust exposure have also been identified in prostate cancer tissues, involving genes in key oncogenic pathways [56]. Mice treated with WTC particulate matter developed an increased burden of mutations in hematopoietic stem and progenitor cell compartments, and WTC-exposed responders developed a significantly higher rate of clonal hematologic abnormalities compared with non-WTC-exposed firefighters [57]. Wu et al. showed that WTC dust may be one of the key etiological factors for those who had been exposed for the development of multiple myeloma by activating mdig and c-myc signaling circuits linked to the IL-6-JAK-STAT3 pathway essential for the tumorigenesis of the malignant plasma cells [58]. These studies highlight the importance of site- and lineage-specific surveillance and mechanistic studies including epigenetic mechanisms to better characterize exposure-associated risks and disease progression in this population. Additionally, exposure to PFAS also carries a well-known risk for cancer, and we and others have shown PFAS are detectable in WTC-exposed community members [19].

This study has several strengths and limitations. The strengths of the WTC EHC program include a demographically diverse civilian population with respect to sex, race, and ethnicity that enables investigation of both common and rare cancers. Limitations include the self-referred nature of the cohort, which introduces potential selection and recall bias, and the restriction to cancers designated as certifiable by the WTC Health Program. Consequently, cancer incidence, prevalence, and mortality rates cannot be directly estimated in this population. There is the potential for increased identification of cancers due to the screening policies of the WTC Health Program, which follows recommendations of the United States Preventive Services Task Force, for screening for breast cancer, cervical cancer, colorectal cancer, and lung cancer. WTC EHC patients are educated about these screenings and offered these screenings during their monitoring examinations. Direct comparisons with regular populations in cancer distribution may be severely influenced by selection bias, differences in healthcare utilization, and enhanced medical surveillance within this cohort. Such comparisons should be interpreted with caution and not as evidence of causal relationships with WTC dust exposure.

Although some cancer types including urinary bladder and melanoma of the skin increased in this cohort compared with our previous report, direct comparison with U.S. population-based estimates such as SEER should be interpreted cautiously. The WTC survivor cohort represents a selected and intensively monitored population that differs from the general U.S. population with respect to exposure history, healthcare utilization, and screening practices. Because this descriptive study was not designed to calculate standardized incidence ratios or perform age- and sex-matched comparisons with national cancer incidence data, observed differences cannot be interpreted as evidence of a true increase in cancer risk attributable to WTC exposure.

This data provides a foundation for future studies of solid tumors and lymphoproliferative and hematopoietic malignancies among WTC survivors, including evaluations of environmental exposure-related risk, tumor characteristics, sex-specific cancers, latency, mechanisms of carcinogenesis, and cancer biomarkers. Continued longitudinal follow-up of this population is warranted to better characterize cancer development and prognosis.

## 5. Conclusions

These findings reveal an evolving and heterogeneous cancer profile among community members exposed to the World Trade Center disaster. The results underscore the need for continued surveillance and highlight the importance of tailored prevention and survivorship strategies for this population. The rapid increase in cancer diagnoses emphasizes the importance of screening and close monitoring for cancer development. Study of this demographically diverse community may provide critical insights to inform future, cancer-specific investigations in this unique population.

## Figures and Tables

**Table 1:** Characteristics of patients with/without cancers at enrollment in the WTC EHC as of 31 December 2024 ^#^.

	Level	Overall	Non-Cancer	Cancer
n		16,763	10,175	6588
Sex, n (%)	Female	8425 (50.3)	5295 (52.1)	3130 (47.5)
	Male	8318 (49.7)	4861 (47.9)	3457 (52.5)
Age on 9/11 (years) (median [range])		43.0 [0.0, 89.0]	41.0 [0.0, 89.0]	45.0 [0.0, 81.0]
Race/Ethnicity (%)	Hispanic	3222 (19.2)	2498 (24.6)	724 (11.0)
	NH *-White	6657 (39.8)	3625 (35.7)	3032 (46.0)
	NH *-Black	3302 (19.7)	2065 (20.3)	1237 (18.8)
	Asian	2314 (13.8)	1435 (14.1)	879 (13.3)
	Native American	7 (0.0)	5 (0.0)	2 (0.0)
	Other/Unknown	1237 (7.4)	523 (5.2)	714 (10.8)
BMI **, n (%)	Normal weight (<25)	2986 (30.7)	1787 (31.1)	1199 (30.2)
	Overweight (25–30)	3475 (35.8)	2007 (34.9)	1468 (37.0)
	Obese (≥30)	3257 (33.5)	1958 (34.0)	1299 (32.8)
Individual Income, n (%)	Equal or less than $30,000/year	7075 (50.5)	4522 (54.9)	2553 (44.3)
	More than $30,000/year	6591 (47.1)	3718 (45.1)	2873 (49.9)
	Do not know/Refused	331 (2.4)	0 (0.0)	331 (5.7)
Education, n (%)	Equal or less than high school	4403 (29.3)	2830 (30.8)	1573 (27.0)
	More than high school	10,628 (70.7)	6367 (69.2)	4261 (73.0)
	No Answer/Never Record/Refused	7 (0.0)	5 (0.1)	2 (0.0)
Exposure information				
Dust cloud exposure, n (%)	No	7721 (48.2)	4734 (47.0)	2987 (50.3)
	Yes	8299 (51.8)	5346 (53.0)	2953 (49.7)
Smoking status (%)	Current smoker	1323 (8.2)	1014 (10.0)	309 (5.2)
	Former smoker	4537 (28.1)	2639 (25.9)	1898 (31.8)
	Never smoker	10,137 (62.8)	6421 (63.1)	3716 (62.3)
	Unknown	143 (0.9)	101 (1.0)	42 (0.7)
Smoking pack year, n (%)	≤5 p-y ***	10,243 (71.8)	6468 (75.1)	3775 (66.8)
	>5 p-y ***	4014 (28.2)	2140 (24.9)	1874 (33.2)

^#^ Only available data is reported for each variable. * NH: non-Hispanic, ** the unit for BMI is kg/m^2^, *** p-y: pack year.

**Table 2:** The median age of diagnosis and median latency in years from 9/11 distribution for top fifteen cancer diagnoses in male and female patients in the WTC EHC.

	All (6983)			Male (3675)			Female (3308)		
	n (%)	Age at Diagnosis (Median [IQR])	Latency Year (Median [IQR])	n (%)	Age at Diagnosis (Median [IQR])	Latency Year (Median [IQR])	n (%)	Age at Diagnosis (Median [IQR])	Latency Year (Median [IQR])
Breast	1677 (24.0)	56.8 [49.3, 64.7]	14.4 [10.2, 17.8]	22 (0.6)	63.6 [52.6, 66.6]	15.8 [10.0, 16.6]	1655 (50.0)	56.7 [49.2, 64.6]	14.4 [10.2, 17.8]
Prostate	1424 (20.4)	63.4 [58.1, 69.1]	15.0 [10.8, 18.5]	1424 (38.7)	63.4 [58.1, 69.1]	15.0 [10.8, 18.5]	-	-	-
Lung	607 (8.7)	65.9 [59.6, 72.1]	16.1 [12.8, 19.1]	258 (7.0)	66.9 [61.5, 73.1]	16.0 [12.8, 19.1]	349 (10.6)	64.8 [58.4, 71.3]	16.1 [12.9, 19.0]
Thyroid	474 (6.8)	52.2 [44.5, 60.4]	13.4 [8.8, 16.9]	181 (4.9)	53.0 [45.3, 61.9]	13.6 [8.5, 17.0]	293 (8.9)	51.5 [43.1, 58.6]	13.2 [8.9, 16.8]
Lymphoma	471 (6.7)	58.4 [49.7, 66.2]	13.8 [9.0, 17.1]	302 (8.2)	58.0 [49.5, 66.1]	13.8 [8.7, 17.1]	169 (5.1)	59.5 [50.0, 66.2]	13.8 [9.3, 17.3]
Colon and rectum	448 (6.4)	59.9 [52.2, 68.4]	15.1 [11.8, 18.5]	290 (7.9)	59.7 [52.0, 68.5]	15.6 [12.3, 18.8]	158 (4.8)	60.2 [52.6, 68.3]	14.6 [10.9, 18.1]
Melanoma of the skin	313 (4.5)	62.2 [52.7, 70.6]	14.3 [10.0, 18.3]	215 (5.9)	63.5 [54.4, 70.9]	14.2 [10.3, 17.8]	98 (3.0)	59.3 [47.0, 69.4]	14.6 [9.3, 18.8]
Head and neck sites	294 (4.2)	59.5 [52.3, 66.6]	14.0 [10.3, 17.7]	225 (6.1)	59.5 [52.8, 67.3]	14.1 [9.8, 17.8]	69 (2.1)	59.5 [51.5, 64.4]	13.6 [11.3, 16.8]
Kidney	276 (4.0)	60.6 [51.2, 67.9]	15.2 [11.5, 18.5]	202 (5.5)	61.0 [52.3, 67.9]	15.1 [11.5, 18.5]	74 (2.2)	59.2 [46.8, 67.8]	15.5 [10.7, 18.5]
Urinary bladder	225 (3.2)	65.9 [59.1, 71.3]	15.1 [11.0, 18.0]	184 (5.0)	65.7 [59.3, 70.7]	15.1 [10.8, 18.0]	41 (1.2)	66.3 [56.8, 74.7]	14.4 [11.8, 17.9]
Leukemia	212 (3.0)	61.4 [51.3, 67.6]	13.9 [9.9, 16.8]	142 (3.9)	61.3 [51.3, 66.9]	12.9 [9.6, 16.0]	70 (2.1)	61.6 [52.1, 68.1]	15.9 [12.5, 18.8]
Myeloma	210 (3.0)	61.2 [54.8, 67.6]	15.4 [12.2, 18.4]	122 (3.3)	61.8 [56.4, 68.1]	15.3 [11.7, 18.3]	88 (2.7)	60.2 [51.1, 67.0]	15.7 [12.5, 18.4]
Corpus uteri	167 (2.4)	60.8 [55.2, 66.5]	15.3 [11.4, 18.9]	-	-	-	167 (5.0)	60.8 [55.2, 66.5]	15.3 [11.4, 18.9]
Pancreas	96 (1.4)	63.2 [54.8, 70.8]	16.2 [12.6, 19.1]	53 (1.4)	66.6 [55.6, 72.6]	16.6 [12.6, 19.6]	43 (1.3)	60.3 [51.1, 67.2]	16.1 [13.2, 18.5]
Stomach	84 (1.2)	58.9 [53.0, 66.8]	14.3 [11.8, 16.9]	52 (1.4)	58.0 [52.6, 65.7]	13.9 [11.7, 16.1]	32 (1.0)	59.6 [54.1, 68.8]	14.8 [11.9, 20.2]

**Table 3:** Comparison of the distribution of number of primary cancers diagnoses per cancer patient in the WTC EHC between 31 December 2019 and 31 December 2024.

	Year 2024		Year 2019	
Number of Cancers	n	%	n	%
1	5644	85.7	2256	86.9
2	842	12.8	299	11.7
3 or more	102	1.5	36	1.4
Total	6588	100	2561	100

**Table 4:** Distribution of the multiple cancers stratified with sex, age at diagnosis of first cancer, and smoking.

	Sex *		Age at First Diagnosis *		Smoking *		Overall
Number of Cancers	Female	Male	<50	>=50	Ever Smoker	Never Smoker	All Patients
1	2699 (86.2%)	2944 (85.2%)	1173 (87.7%)	4416 (85.0%)	1831 (83.0%)	3257 (87.6%)	5644 (85.7%)
2	382 (12.2%)	460 (13.3%)	152 (11.4%)	688 (13.2%)	332 (15.0%)	416 (11.2%)	842 (12.8%)
3 or more	49 (1.6%)	53 (1.5%)	12 (0.9%)	90 (1.7%)	44 (2.0%)	43 (1.2%)	102 (1.5%)
Total patients	3130	3457	1337	5194	2207	3716	6588

* Only available data is reported for each variable.

## Data Availability

The datasets in the WTC EHC pan-cancer database are not publicly available, but de-identified and anonymized information is potentially available upon reasonable request.

## Supplementary Materials

The following supporting information can be downloaded at: https://www.mdpi.com/article/10.3390/ijerph23050625/s1. Table S1: The median age of diagnosis and median latency from 9/11 for non-common cancers in both sexes. Table S2: Distribution of the percentage of the solid and lymphoproliferative/hematopoietic malignancies in the WTC EHC through 31 December 2020.
