# Supplementary material for: Evolving Cancer Characteristics Among World Trade Center Survivors: An Updated Analysis from the WTC Environmental Health Center
Source: Int J Environ Res Public Health. 2026 May 8;23(5):625. doi: 10.3390/ijerph23050625 (PMC13205631; doi:10.3390/ijerph23050625)
Supplement: Supplementary file 1 [file ijerph-23-00625-s001.zip › ijerph-4192557-supplementary.pdf]

Supplementary Table S1. The median age of diagnosis and median latency in years from 9/11 distribution for less common cancer diagnoses in male and female patients in the WTC EHC*.

	All			Male			Female		
	n (%)	Age at diagnosis (median [IQR])	Latency year (median [IQR])	n (%)	Age at diagnosis (median [IQR])	Latency year (median [IQR])	n (%)	Age at diagnosis (median [IQR])	Latency year (median [IQR])
Ovary	77 (12.8)	57.2 [50.0, 66.1]	13.9 [11.8, 18.1]	-	-	-	77 (28.7)	57.2 [50.0, 66.1]	13.9 [11.8, 18.1]
Brain and spinal cord	75 (12.5)	55.6 [44.6, 62.8]	13.8 [10.4, 16.9]	37 (11.1)	53.6 [45.4, 62.6]	13.3 [10.7, 16.5]	38 (14.2)	56.0 [43.9, 67.1]	14.2 [10.1, 17.3]
Soft tissue sarcoma	66 (11.0)	56.4 [49.5, 64.1]	16.2 [11.9, 18.6]	38 (11.4)	55.7 [49.5, 64.3]	16.0 [11.8, 18.9]	28 (10.4)	57.0 [49.1, 61.8]	16.3 [12.8, 17.9]
Liver	64 (10.6)	62.7 [58.1, 70.4]	14.8 [12.5, 19.2]	47(14.1)	62.7 [59.9, 70.6]	15.3 [13.2, 19.2]	17 (6.3)	61.9 [55.1, 67.2]	14.0 [12.3, 18.0]
Esophagus	59 (9.8)	60.2 [53.9, 66.3]	16.0 [11.6, 19.4]	51 (15.3)	60.2 [53.9, 66.3]	16.0 [12.0, 19.4]	8 (3.0)	61.4 [54.6, 67.0]	14.2 [10.6, 18.0]
Myeloproliferative neoplasm	50 (8.3)	60.0 [53.8, 68.7]	13.8 [9.8, 17.9]	33 (9.9)	60.0 [54.4, 69.9]	14.0 [10.8, 18.4]	17 (6.3)	59.6 [46.6, 68.7]	10.1 [5.7, 16.7]
Testis	46 (7.7)	40.9 [33.9, 47.6]	10.7 [6.2, 15.0]	46 (13.8)	40.9 [33.9, 47.6]	10.7 [6.2, 15.0]	-	-	-
Myelodysplastic syndrome	35 (5.8)	65.5 [55.6, 73.1]	15.6 [13.3, 19.5]	22 (6.6)	65.4 [56.6, 70.4]	14.9 [13.4, 18.5]	13 (4.9)	69.6 [49.4, 74.1]	15.9 [13.3, 19.8]
Small Intestine	23 (3.8)	60.1 [53.0, 69.5]	14.4 [8.4, 16.0]	14 (4.2)	61.4 [56.0, 68.0]	15.0 [13.0, 16.0]	9 (3.4)	53.1 [52.2, 69.8]	9.9 [5.8, 14.9]
Cervix uteri	19 (3.2)	53.3 [44.4, 60.0]	14.1 [8.1, 17.3]	-	-	-	19 (7.1)	53.3 [44.4, 60.0]	14.1 [8.1, 17.3]
Neuroendocrine tumor	12 (2.0)	62.9 [56.6, 71.7]	17.8 [15.2, 19.2]	5 (1.5)	53.5 [51.0, 62.8]	15.3 [13.5, 18.9]	7 (2.6)	71.3 [65.2, 73.1]	18.2 [17.0, 19.5]
Soft Tissue Neoplasm	12 (2.0)	55.5 [43.1, 72.1]	16.9 [13.1, 19.9]	7 (2.1)	57.3 [42.1, 72.3]	17.8 [12.1, 20.5]	5 (1.9)	54.8 [54.4, 56.1]	16.3 [14.6, 17.5]
Bone	11 (1.8)	53.3 [41.5, 56.8]	13.5 [7.3, 17.1]	9 (2.7)	53.3 [42.8, 54.9]	13.5 [5.5, 18.3]	2 (0.7)	52.9 [42.7, 63.1]	14.1 [13.5, 14.8]
Ophthalmic site tumor	11 (1.8)	60.0 [44.2, 65.4]	13.0 [8.8, 16.0]	6 (1.8)	63.3 [58.0, 65.6]	13.0 [11.1, 13.8]	5 (1.9)	43.2 [40.6, 60.0]	10.3 [5.8, 17.9]
Vulva	11 (1.8)	58.3 [55.9, 59.0]	15.8 [13.8, 18.5]	-	-	-	11 (4.1)	58.3 [55.9, 59.0]	15.8 [13.8, 18.5]
Extrahepatic bile duct	10 (1.7)	63.2 [56.2, 66.7]	14.8 [13.4, 16.7]	4 (1.2)	65.7 [63.5, 68.8]	19.0 [16.3, 21.5]	6 (2.2)	57.8 [54.3, 62.7]	13.7 [12.6, 14.6]
Thymus	10 (1.7)	57.7 [52.8, 64.1]	13.7 [12.8, 15.1]	6 (1.8)	59.8 [52.0, 64.4]	12.8 [12.2, 13.7]	4 (1.5)	57.6 [56.3, 59.3]	17.8 [14.5, 20.7]
Ureter	10 (1.7)	70.5 [62.0, 75.3]	20.1 [16.7, 20.9]	8 (2.4)	68.2 [57.4, 72.2]	20.1 [14.9, 20.9]	2 (0.7)	78.0 [77.0, 79.0]	20.5 [19.8, 21.2]

*Cancers with fewer than 10 diagnoses were not included in the supplementary table.

Supplemental Table S2. Distribution of the percentage of the solid and lymphoproliferative/hematopoietic malignancies in the WTC EHC through December 31, 2020.

Year group	Cancer group	n	Percentage
up to 2005	Hematologic	55	41.7
	Solid	77	58.3
2006	Hematologic	22	12.0
	Solid	161	88.0
2007	Hematologic	23	10.3
	Solid	201	89.7
2008	Hematologic	40	13.8
	Solid	250	86.2
2009	Hematologic	34	12.0
	Solid	249	88.0
2010	Hematologic	36	11.7
	Solid	273	88.3
2011	Hematologic	43	13.2

	Solid	282	86.8
**2012**	Hematologic	45	12.6
	Solid	313	87.4
**2013**	Hematologic	53	13.4
	Solid	344	86.6
**2014**	Hematologic	64	12.6
	Solid	445	87.4
**2015**	Hematologic	67	12.5
	Solid	470	87.5
**2016**	Hematologic	89	15.2
	Solid	497	84.8
**2017**	Hematologic	68	13.0
	Solid	454	87.0
**2018**	Hematologic	65	11.5
	Solid	498	88.5
**2019**	Hematologic	60	11.1
	Solid	482	88.9
**2020**	Hematologic	70	13.6
	Solid	443	86.4
